# The use of patient‐derived breast tissue explants to study macrophage polarization and the effects of environmental chemical exposure

**DOI:** 10.1111/imcb.12381

**Published:** 2020-09-09

**Authors:** Kelly J Gregory, Stephanie M Morin, Alex Kubosiak, Jennifer Ser‐Dolansky, Benjamin J Schalet, D Joseph Jerry, Sallie S Schneider

**Affiliations:** ^1^ Pioneer Valley Life Sciences Institute Springfield MA 01199 USA; ^2^ Biology Department University of Massachusetts Amherst MA 01003 USA; ^3^ Tulane University New Orleans LA 70118 USA; ^4^ Veterinary and Animal Sciences University of Massachusetts Amherst MA 01003 USA; ^5^ Department of Surgery University of Massachusetts Medical School/Baystate Springfield MA 01199 USA

**Keywords:** EMT, macrophage polarization, oxybenzone, patient‐derived explant

## Abstract

*Ex vivo* mammary explant systems are an excellent model to study interactions between epithelium and stromal cell types because they contain physiologically relevant heterotypic interactions in the background of genetically diverse patients. The intact human mammary tissue, termed patient‐derived explant (PDE), can be used to investigate cellular responses to a wide variety of external stimuli *in situ*. For this study, we examined the impact of cytokines or environmental chemicals on macrophage phenotypes. We demonstrate that we can polarize macrophages within human breast tissue PDEs toward M1 or M2 through the addition of interferon‐γ (IFNγ) + lipopolysaccharide (LPS) or interleukin (IL)‐4 + IL‐13, respectively. Elevated expression levels of M(IFNγ + LPS) markers (*HLADRA* and *CXCL10*) or M(IL‐4 + IL‐13) markers (CD209 and CCL18) were observed in cytokine‐treated tissues. We also examined the impact of the endocrine‐disrupting chemical, benzophenone‐3, on PDEs and measured significant, yet varying effects on macrophage polarization. Furthermore, a subset of the PDEs respond to IL‐4 + IL‐13 through downregulation of E‐cadherin and upregulation of vimentin which is reminiscent of epithelial‐to‐mesenchymal transition (EMT) changes. Finally, we were able to show immortalized nonmalignant breast epithelial cells can exhibit EMT characteristics when exposed to growth factors secreted by M(IL‐4 + IL‐13) macrophages. Taken together, the PDE model system is an outstanding preclinical model to study early tissue‐resident immune responses and effects on epithelial and stromal responses to stimuli found both endogenously in the breast and exogenously as a result of exposures.

## Graphical abstract

The intact human mammary tissue, termed patient‐derived explant (PDE), can be used to investigate cellular responses to a wide variety external stimuli *in situ*. For this study, we examined the impact of cytokines or environmental chemicals on macrophage phenotypes. We demonstrate that we can polarize macrophages within human breast tissue PDEs toward M1 or M2 and the endocrine‐disrupting chemical, benzophenone‐3, also affects macrophage polarization.

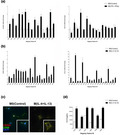

## Introduction

Macrophages comprise a portion of immune cells that are phagocytic in nature and are present in almost all tissues. In the breast, macrophages play an important role in ductal/lobular development.^1^ In addition, macrophages are well established constituents of the breast tumor microenvironment as evidenced by the finding that increased macrophage density in biopsies of breast cancer patients correlates with reduced recurrence‐free and overall survival.^2^, ^3^, ^4^ Depending on the type of signal in the microenvironment, macrophages are polarized into distinct phenotypes. Classically activated M1 macrophages are involved in T helper type 1 responses to pathogens and play an important role in both innate host defenses and tumoricidal activities by expressing proinflammatory cytokines. Therefore, they are considered to be antitumor macrophages.^5^ Alternatively activated M2 macrophages are induced by T helper type 2 cytokines and are generally divided into four subtypes critical for wound healing (M2a), immunoregulation (M2b), immunosuppression (M2c) and tumor development (M2‐like/M2d).^6^ In this case, M2 macrophages are largely considered to be protumor macrophages.^7^ Tumor‐associated macrophages (TAMs) are the most abundant population of tumor‐infiltrating immune cells in the tumor microenvironment and can consist of both M1 and M2 macrophages.^8^ However, TAMs are more frequently observed to be of the M2 type and secrete soluble factors that typically facilitate angiogenesis, tumor initiation, growth and metastasis.^3^

Epithelial‐to‐mesenchymal transitions (EMTs) are involved in the transformation of early‐stage tumors into invasive cancers. During EMTs epithelial cells lose polarity and the cytoskeleton is rearranged such that cell–cell contacts are lost and a more mesenchymal morphology is taken on. Downregulation of E‐cadherin (CDH), a fundamental constituent of cell–cell adhesion junctions, is a hallmark of EMT.^9^ In addition, upregulation of vimentin (VIM), a type 3 intermediate filament that is commonly expressed in mesenchymal cells and migratory epithelial cells, is observed.^10^ It has been reported that TAMs are associated with EMT and that cytokines secreted by TAMs in the tumor microenvironment promote the EMT of a multitude of cancer cell types, including breast cancer cells.^11^, ^12^, ^13^, ^14^, ^15^, ^16^ For example, M2‐polarized macrophages have been shown to induce a mesenchymal phenotype in MCF‐7 breast cancer cells and also repress the expression of E‐cadherin.^17^ However, until now little has been understood about how M2‐polarizing cytokines affect nonmalignant breast epithelial cells.

Using immortalized human cell line models *in vitro* or *in vivo* has aided researchers in the understanding of how epithelial cells respond to neighboring cell signals. Ideally, the most advantageous approach would be to evaluate how cellular responses to external stimuli *in situ* affect cells that have normal heterotypic interactions. An *ex vivo* model system composed of intact human breast tumor tissue, which is termed patient‐derived explant (PDE), has been described.^18^ Breast tumor PDEs retain many features of human solid tumors including the native microenvironment and cellular interactions that are required for tumor formation. This approach is stable as evidenced by sustained tissue morphology, viability and the maintenance of intact cellular signaling networks.^18^ As such, this model system has become a preeminent model for investigating preclinical interventions with respect to cancer treatment. By contrast, normal human breast and murine mammary explant culture systems have been employed to investigate how various treatments affect cell signaling pathways.^19^, ^20^, ^21^, ^22^ Moreover, it has been shown that macrophages within murine epididymal adipose tissue cultured *ex vivo* can be polarized toward M1 or M2 phenotypes when stimulated with T helper type 1 or T helper type 2 cytokines, respectively.^23^

For this study, we utilized the human benign breast PDE culture system to demonstrate that polarizing cytokines affect canonical M1 and M2 markers within intact human breast glandular tissue, which in turn alters EMT‐associated gene expression. In addition, because endocrine‐disrupting chemicals have been shown to affect macrophage polarization,^24^ we utilized the PDE culture system and revealed that benzophenone‐3 (BP3), the main ingredient in sunscreen,^25^ increases the expression of genes associated with M2 macrophage polarization in a subset of patients. Taken together, our results highlight an outstanding model system to elucidate how macrophages from different patients respond to external stimuli.

## Results

### PDEs treated with polarizing agents increase the expression of markers consistent with polarized myeloid cells

**Figure 1:**
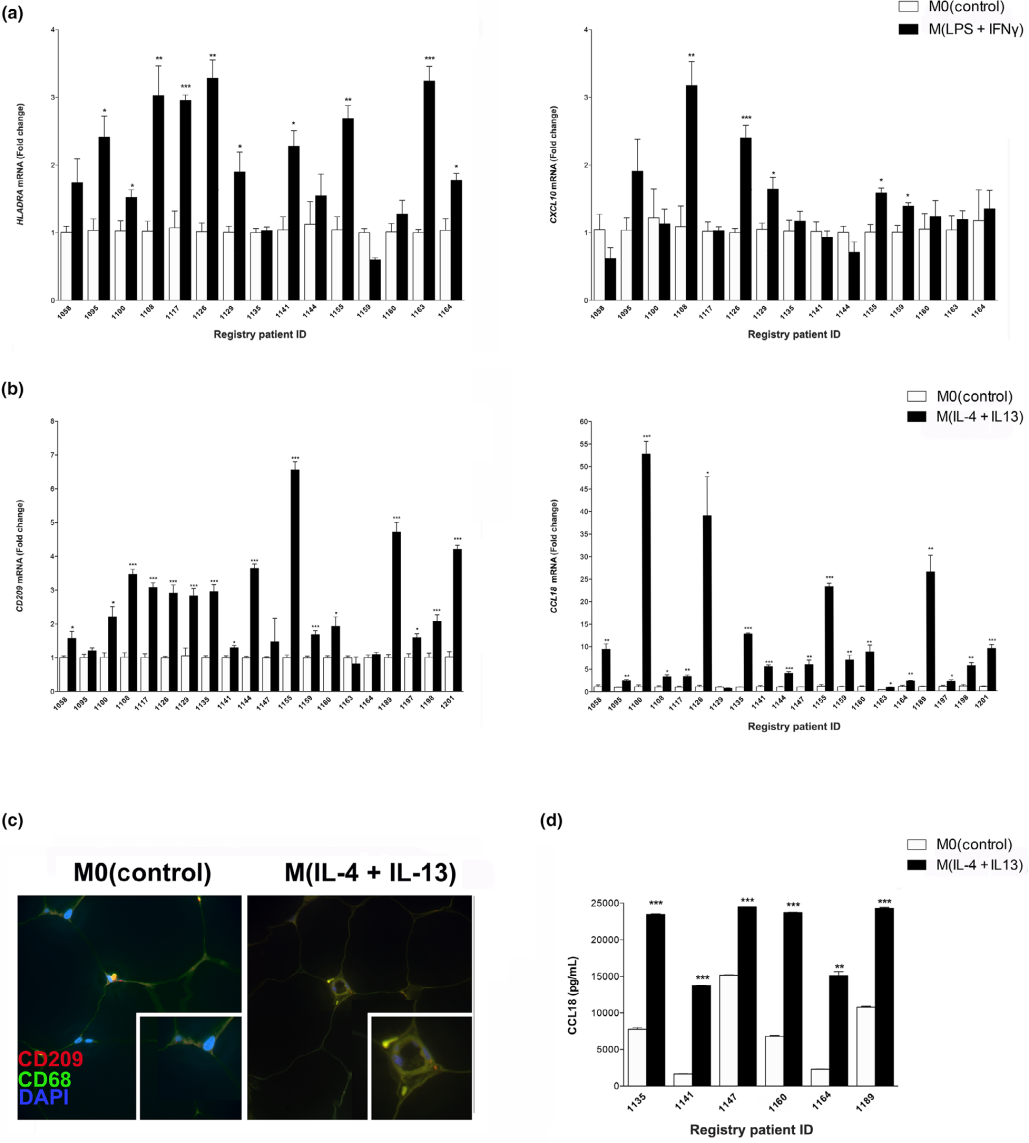
Normal breast PDEs can be polarized toward M(IFNγ + LPS) or M(IL‐4 + IL‐13). RNA was harvested from cytokine‐exposed PDEs and mRNA levels of **(a)** *HLA‐DRA* and *CXCL10* (15 patients) or **(b)** *CD209* and *CCL18* (20 patients) were analyzed by real‐time PCR. All real‐time PCR results are from two separate experiments (performed technical duplicate) and results were normalized to amplification of *CD68* (macrophage marker). **(c)** PDE sections were subjected to fluorescent immunohistochemical analysis, dual stained for CD68 and CD209 and merged images were captured at 4000×. Representative pictures are displayed for tissues from each treatment group. **(d)** Supernatant was collected from PDEs (six patients) treated with IL‐4 + IL‐13 cytokines and CCL18 protein secretion was measured by ELISA performed in biological triplicate and technical duplicate M0(control) PDEs. Data within each bar represent triplicate samples isolated from individual patients, are presented as mean ± s.e.m. and are expressed as fold change with respect to M0(control) PDEs. *P < 0.05, **P< 0.01, ***P< 0.001 (significantly different from indicated data set using a Student’s *t*‐test). IFN, interferon; IL, interleukin; LPS, lipopolysaccharide; mRNA, messenger RNA; PDE, patient‐derived explant.

Fresh human benign breast tissues were set up as PDEs and the media were left untreated, treated with M1‐polarizing cytokines [lipopolysaccharide (LPS) + interferon‐γ (IFNγ)] or treated with M2a‐polarizing cytokines [interleukin (IL)‐4 + IL‐13] for 3 days. Medical history from each patient is listed in Table 1. Although the epithelial cellular integrity of PDEs after 3 days in culture has previously been described,^18^ we sought to determine whether immune cell viability was affected by our treatment strategy. The number of macrophages (CD68‐positive cells) and T cells (CD3‐positive cells) was counted before and after 3 days in culture. We found that neither the number of macrophages nor the number T cells were affected by our PDE culture assays (Supplementary figure 1a). Furthermore, because it has been shown that macrophages stimulated with LPS and IFNγ exhibit a decrease in viability over time,^26^, ^27^ we examined the effect of these cytokines on genes typically activated in death pathways. We show that the expression levels of *BAX, BIM* and *HIF1A* are not altered by 72 h in explant culture in response to LPS + IFNγ treatment (Supplementary figure 1b). The markers consistent with M(IFNγ + LPS) and M(IL‐4 + IL‐13) polarization were selected based on evaluation of six different cell surface and secreted proteins for each subtype (Supplementary figure 2). To assess the effect of IFNγ and LPS on changes consistent with M1‐type polarization within breast tissues, RNA was harvested from 15 cytokine‐exposed PDEs and messenger RNA (mRNA) levels of *HLA‐DRA* and *CXCL10* (M1 markers) were measured using real‐time PCR and results were normalized to total macrophage expression (*CD68*; Figure 1a). It has been previously established that HLA‐DRA is a cell surface protein expressed on M(IFNγ + LPS)‐polarized macrophages^28^ and here it was revealed that *HLA‐DRA* mRNA expression is significantly elevated in 10 of 15 patient tissues treated with LPS and IFNγ. Furthermore, CXCL10 is secreted by M(IFNγ + LPS) macrophages^29^ and *CXCL10* expression is significantly elevated in 5 of 15 cytokine‐treated PDEs. A Wilcoxon signed‐rank test indicated that LPS + IFNγ‐stimulated PDEs expressed statistically significantly higher *HLADRA* and *CXCL10* mRNA levels than untreated PDEs (*P* = 0.0002, *P* = 0.0177). When we treated PDEs with IL‐4 + IL‐13, 16 of 20 patients exhibited a significant increase in *CD209 (DC‐SIGN),* a cell surface lectin found on M(IL‐4 + IL‐13)‐polarized macrophages and monocyte‐derived dendritic cells.^30^, ^31^ A Wilcoxon signed‐rank test indicated that IL‐4 + IL‐13‐stimulated PDEs expressed statistically significantly higher *CD209* mRNA levels than untreated PDEs (*P* < 0.0001). Dual immunofluorescent staining with antibodies specific to CD209 and CD68 in paraffin‐embedded tissues was performed on untreated and IL‐4 + IL‐13‐treated PDEs. The merged image shows that CD209 is not expressed in untreated PDEs and as such only the CD68 stain is observed. PDEs treated with IL‐4 + IL‐13 demonstrate the localization of CD209 within CD68‐positive cells (Figure 1c). Furthermore, we carried out immunohistochemistry for CD68 to evaluate the number of macrophages. Linear regression analysis revealed that the number CD68‐stained cells was not correlated with the fold increase in CD209 (*r* ^2^ = 0.0708; *P* = 0.3375) or CCL18 (*r* ^2^ = 0.1142; *P* = 0.1627) expression among patients (Supplementary figure 3), which supports the notion that the increase in M(IL‐4 + IL‐13) marker expression is not driven by an increase in the number of macrophages. Furthermore, we next measured CCL18, an IL‐4‐induced chemokine important for attracting T cells and for polarization of M2 macrophages,^32^ in M(IL‐4 + IL‐13)‐treated PDEs. Our data demonstrate that IL‐4 + IL‐13 increase the expression of CCL18 in 19 of 20 treated PDEs (Figure 1b). A Wilcoxon signed‐rank test indicated that IL‐4 + IL‐13‐stimulated PDEs expressed statistically significantly higher *CCL18* mRNA levels than untreated PDEs (*P* < 0.0001). The medium from six PDE cultures was collected and ELISAs confirmed that CCL18 secretion is significantly elevated in all samples collected (Figure 1d). A Wilcoxon signed‐rank test indicated that IL‐4 + IL‐13‐stimulated PDEs expressed statistically significantly higher *CCL18* protein levels than untreated PDEs (*P* = 0.0156).

**Table 1:** Demographic information of Rays of Hope Registry patients utilized for PDE cultures.

Patient ID	Age (years)	BMI (kg/m^2^)	Parity	Surgery type	History of cancer	Cancer near PDE	Ethnicity
1058	20	27	Nulliparous	Reduction	No	No	White
1095	22	30	Nulliparous	Reduction	No	No	White
1100	65	36	Nulliparous	Reduction	No	No	White
1117	64	20	Parous	Prophylactic bilateral mastectomy	No	No	White
1126	57	28	Nulliparous	Unilateral mastectomy	Yes	Yes	White
1129	56	34	Parous	Unilateral mastectomy	Yes	Yes	White
1135	54	21	Parous	Unilateral mastectomy	No	No	White
1141	67	31	Parous	Unilateral mastectomy	Yes	Yes	White
1144	60	32	Parous	Reduction	Yes	Yes	Latina/White
1147	49	23	Parous	Reduction	Yes	No	White
1155	57	25	Parous	Reduction	No	No	White
1159	64	27	Parous	Lumpectomy	Yes	Yes	White
1160	51	41	Parous	Oncoplastic reduction	Yes	No	White
1163	41	27	Parous	Bilateral mastectomy	Yes	No	White
1164	60	39	Parous	Oncoplastic reduction	Yes	No	White
1189	55	36	Parous	Unilateral mastectomy	Yes	Yes	White
1197	49	29	Parous	Reduction	No	No	White
1198	18	30	Nulliparous	Reduction	No	No	White
1201	68	29	Parous	Unilateral mastectomy	Yes	Yes	White

BMI, body mass index; PDE, patient‐derived explant.

### Benzophenone‐3‐treated PDEs and primary monocytes polarize macrophages toward an M2a phenotype

**Figure 2:**
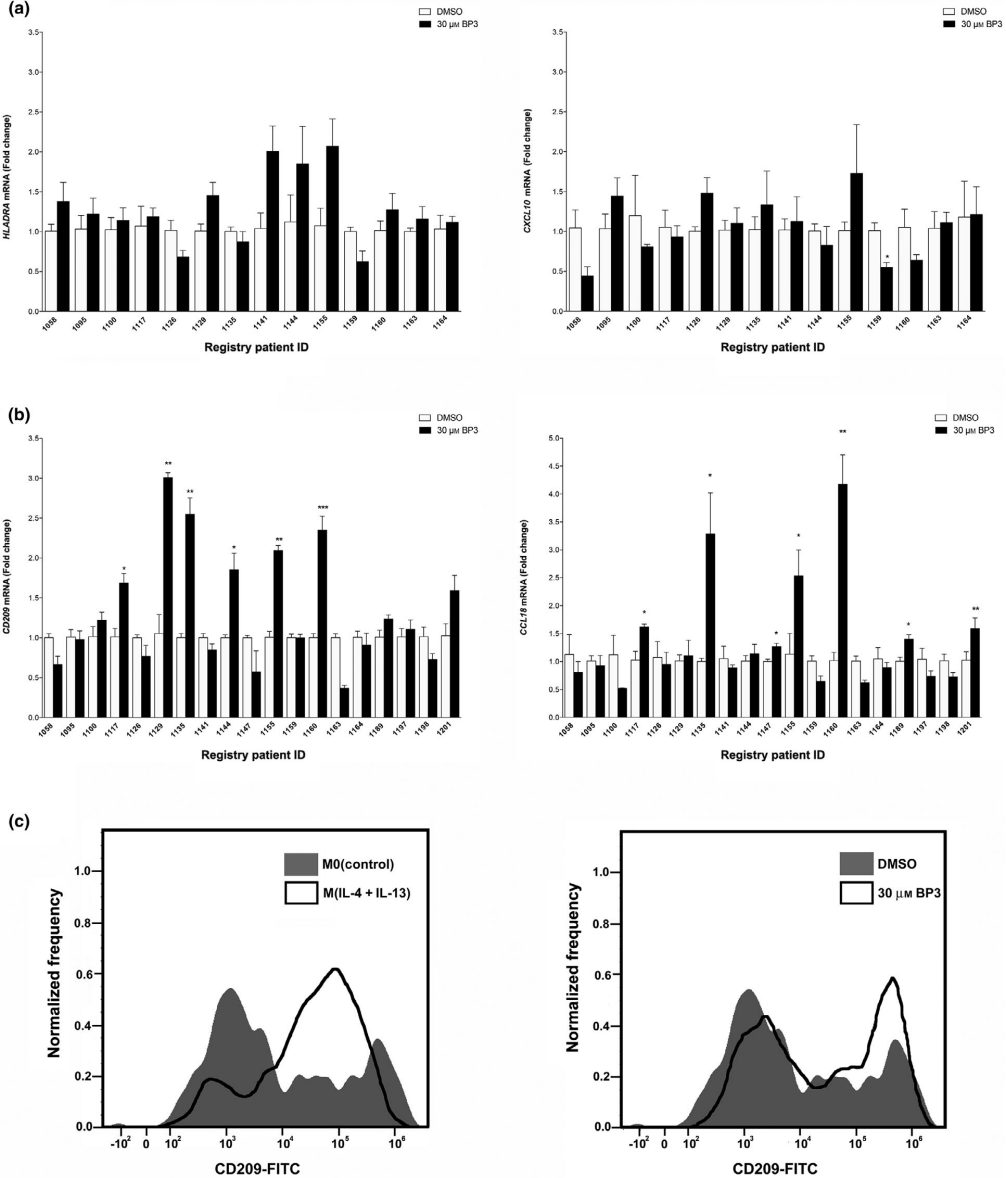
The xenoestrogen, BP3, can increase the expression of M2a markers in a subset of PDEs. RNA was harvested from PDEs treated with either vehicle (DMSO) or 30 μm BP3 and mRNA levels of **(a)** *HLA‐DRA* and *CXL10* (14 patients) or **(b)** *CD209* and *CCL18* (19 patients) were analyzed by real‐time PCR. All real‐time PCR results are from two separate experiments (performed in technical duplicate) and results were normalized to amplification of *CD68* (macrophage marker). Data within each bar represent triplicate samples isolated from individual patients, are presented as mean ± s.e.m. and are expressed as fold change with respect to DMSO treated M0(control) PDEs. *P < 0.05, **P < 0.01, ***P < 0.001 (significantly different from indicated data set using a Student’s *t*‐test). **(c)** Cell surface protein expression of CD209 in treated primary human macrophages was measured by flow cytometry. BP3, benzophenone‐3; DMSO, dimethyl sulfoxide; FITC, fluorescein isothiocyanate; mRNA, messenger RNA; PDE, patient‐derived explant.

We sought to use this model system to examine the impact of environmental exposures on breast tissue. Endocrine‐disrupting chemicals have been demonstrated to affect breast gland development and facilitate cancer growth; however, the epithelium is not the only cell type expressing hormone receptors. Numerous stromal cells, including monocytes and macrophages, express receptors which have been shown to impact immune cell chemotaxis, differentiation and activation.^33^, ^34^ In fact, the endocrine‐disrupting chemical bisphenol A (BPA) has been shown to increase the expression of several M2‐related proteins and facilitate metastatic growth of a ductal carcinoma *in situ* lesion.^24^ We wished to determine whether the endocrine disrupting chemical and UV filter, BP3, could affect gene expression patterns consistent with macrophage polarization in PDEs. RNA was harvested from PDEs treated with either vehicle (dimethyl sulfoxide) or 30 μm BP3 for 3 days and mRNA levels of *HLA‐DRA, CXCL10, CD209* and *CCL18* were analyzed by real‐time PCR (Figure 2). While none of the PDEs treated with BP3 expressed elevated levels of M1 markers (Figure 2a), we reveal that 6 of 19 patients express significantly elevated levels of *CD209* mRNA and 7 of 19 express significantly higher levels of *CCL18* mRNA (Figure 2b) in response to BP3 exposure. Contrastingly, the PDE from one patient (registry patient ID 1163) exhibited a decrease in CD209 and CCL18 in response to BP3 exposure. It is unclear whether this decrease was a result of something different (i.e. microenvironmental cytokines) in the tissues of this patient or if the cellular composition of the tissue treated with BP3 was different. CCL18 also decreased in response to BP3 in the PDE tissue from patient 1159 but there was no effect of BP3 on CD209. Taken together, we suspect that the few instances of decreased M2‐related markers are likely because of variation within the tissue.

To establish the direct effects of BP3 on macrophage polarization, primary macrophages were isolated from human blood samples. To validate M(IL‐4 + IL‐13) polarization, primary human macrophages were stimulated with cytokines for 72 h. CD209 cell surface protein expression was detected via flow cytometry as a marker for M2 macrophage polarization (Figure 2c). Subsequently, macrophages subjected to 3 days of BP3 exposure were collected and flow cytometry data confirmed the increase in the CD209 cell surface protein in response to BP3 (Figure 2c). Real‐time PCR analysis revealed that *CCL18* mRNA was also elevated in primary macrophages in response to BP3 treatment (Supplementary figure 4). Taken together, these data are suggestive of a direct BP3‐induced change altering protein expression consistent with altered polarization.

### M2a‐polarizing cytokines alter EMT‐associated gene expression in normal PDEs and normal breast cells

**Figure 3:**
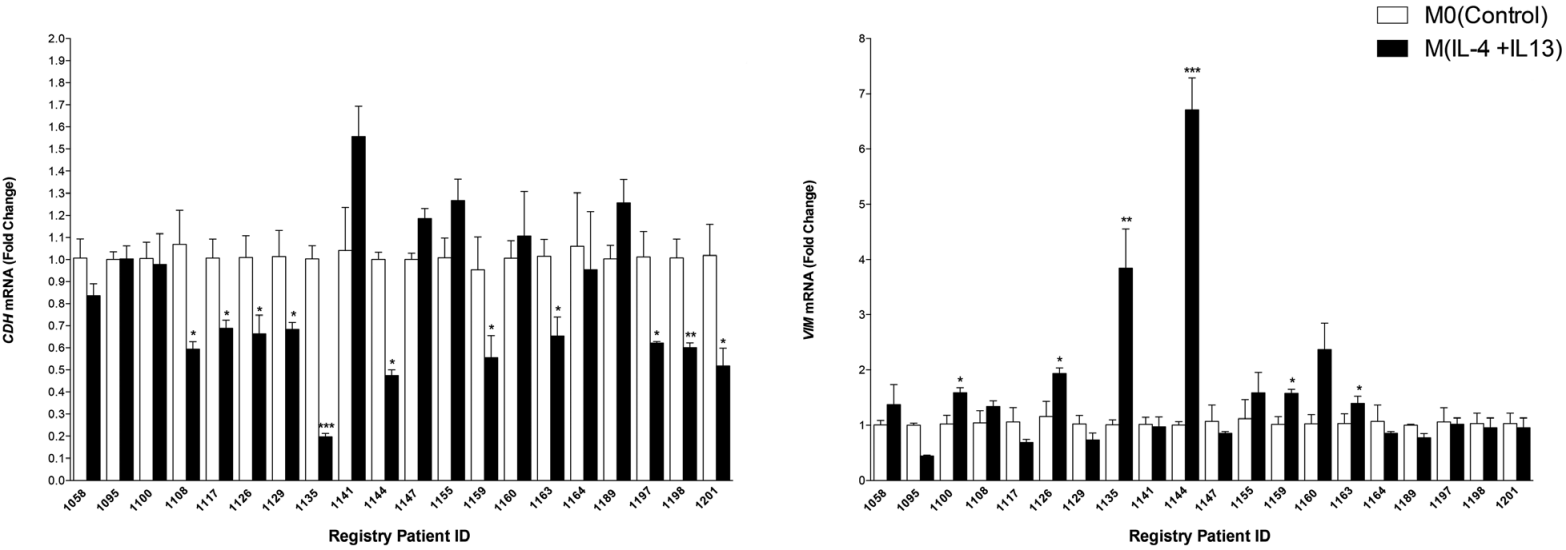
Epithelial cells in PDEs exhibit a gene expression pattern associated with EMT when macrophages are polarized toward M(IL‐4 + IL‐13). RNA was harvested from IL‐4 + IL‐13‐exposed PDEs (20 patients, 72 h) and mRNA levels of **(a)** E‐cadherin *(CDH1)* and **(b)** vimentin (*VIM*) were analyzed by real‐time PCR. All real‐time PCR results are from two separate experiments (performed in technical duplicate) and results were normalized to amplification of CK18 (epithelial cell marker). Data within each bar represent triplicate samples isolated from individual patients, are presented as mean ± s.e.m. and are expressed as fold change with respect to M0(control) PDEs. *P < 0.05, **P < 0.001 ***P < 0.001 (significantly different from indicated data set using a Student’s *t*‐test). EMT, epithelial‐to‐mesenchymal transition; IL, interleukin; mRNA, messenger RNA; PDE, patient‐derived explant.

EMT is an important mechanism employed during development as well as wounding healing to obtain proper movement and placement of cells. This process involves the downregulation of critical adhesion proteins and upregulation of the intermediate filament protein, VIM, which is a marker of mesenchymal‐like cells. Conditioned medium (CM) from M2‐polarized cells has previously been shown to cause EMT and invasion of tumor cells. However, we were interested in whether IL‐4 + IL‐13 would decrease E‐cadherin (CDH1) and increase VIM in normal noncancerous MCF10A mammary epithelial cells and PDE tissues. We measured the mRNA expression of *CDH* and *VIM* to evaluate whether the IL‐4 + IL13‐exposed PDEs would affect the expression of these EMT‐associated genes (Figure 3). Our data reveal that 11 of 20 PDEs treated with M2a‐polarizing cytokines express significantly lower levels of *CDH* gene expression (Figure 3a) and the mRNA expression of *VIM* is significantly increased in 6 of 20 patients (Figure 3b). A Wilcoxon signed‐rank test indicated that IL‐4 + IL‐13‐stimulated PDEs expressed statistically significantly higher *CDH1* mRNA levels than untreated PDEs (*P* = 0.0096) and an increase in VIM was nearly statistically significant (*P* = 0.0604). The finding that only a portion of PDEs exhibited gene expression changes associated with EMT is likely because of the amount of epithelial tissue present in the *ex vivo* tissues.

**Figure 4:**
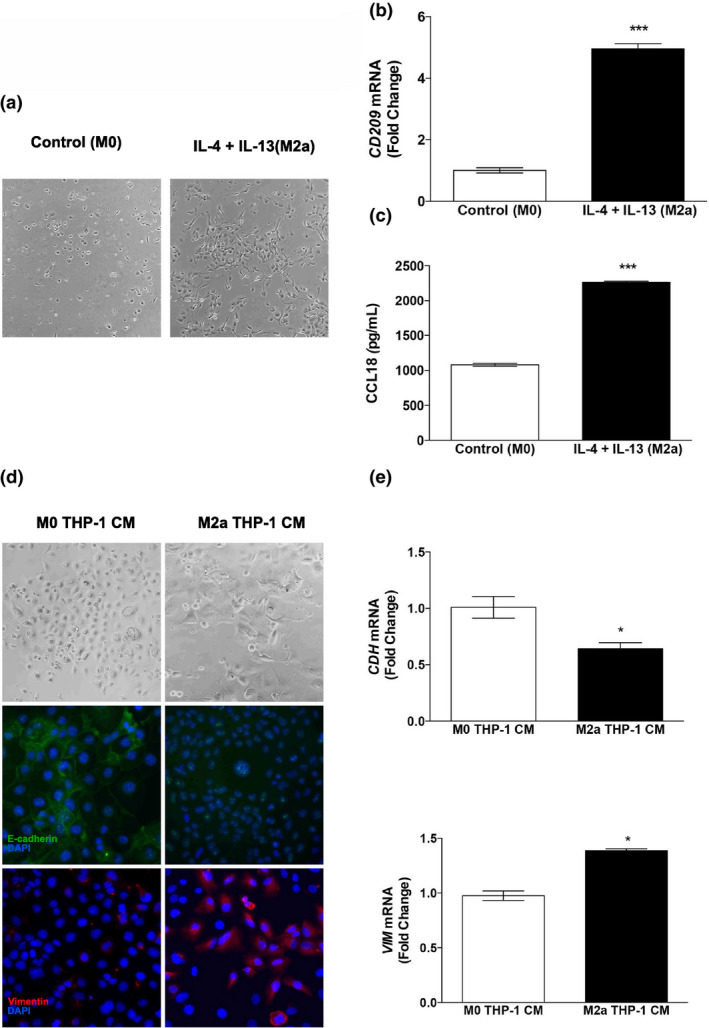
*In vitro* polarization of THP‐1 cells toward the M(IL‐4 + IL‐13) phenotype and the effects of THP‐1 CM on EMT in MCF10A cells. THP‐1 monocytes were treated with a combination of PMA, IL‐4 and IL‐13 and **(a)** images were captured to illustrate the morphological changes in response to cytokine treatment. **(b)** *CD209* mRNA was quantified by real‐time PCR and **(c)** CCL18 secretion in cell culture medium was measured by ELISA in technical duplicates. MCF10A cells treated with THP‐1 CM were grown to 50% confluence and **(d)** images were captured to illustrate the morphological changes in response to THP‐1 CM treatment. The cells were then immunostained with either anti‐E‐cadherin or anti‐vimentin antibodies and counterstained with DAPI. **(e)** RNA was harvested from THP‐1 CM‐treated cells and real‐time PCR analysis of *CDH* and *VIM* was carried out. Data within each bar represent triplicate treatments, are presented as mean ± s.e.m. and are expressed as fold change with respect to controls. *P < 0.05, ***P < 0.001 (significantly different from indicated data set using a Student’s *t*‐test). All images were captured at 400× magnification. CM, conditioned medium; DAPI, 4′,6‐diamidino‐2‐phenylindole; EMT, epithelial‐to‐mesenchymal transition; IL, interleukin; mRNA, messenger RNA; PMA, phorbol 12‐myristate 13‐acetate.

Therefore, we next wished to further investigate the role of IL‐4 + IL‐13‐induced macrophage polarization in affecting EMT in nonmalignant epithelial cells using a simplified *in vitro* system. The human monocytoid cell line (THP‐1) can be polarized toward M2 by differentiation with phorbol 12‐myristate 13‐acetate and subsequent treatment with IL‐4 + IL‐13.^35^ We carried out the same 3‐day treatment protocol utilized for our *ex vivo* PDE studies and polarized THP‐1 monocytes. The morphology of IL‐4 + IL‐13‐treated THP‐1 cells changed to elongated consistent with previously published findings^36^ (Figure 4a). Moreover, real‐time PCR analysis of *CD209* confirmed a significant increase in mRNA expression (Figure 4b) and ELISAs established that CCL18 protein secretion was significantly elevated in response to M2a‐polarizing cytokines (Figure 4c). To confirm potential communication between M2a‐polarized THP1 and nonmalignant breast epithelial cells, CM from unpolarized (M0 THP‐1 CM) and M2‐polarized [M(IL‐4 + IL‐13) THP‐1 CM] cytokines was collected and used to treat MCF10A cells. M(IL‐4 + IL‐13) THP‐1 CM induced a spindle‐like morphology in MCF10A cells compared with M0 THP‐1 CM (Figure 4e). Immunocytochemistry and real‐time PCR analyses demonstrated that protein and mRNA levels of CDH1 were significantly reduced and VIM levels were significantly elevated (Figure 4e).

**Figure 5:**
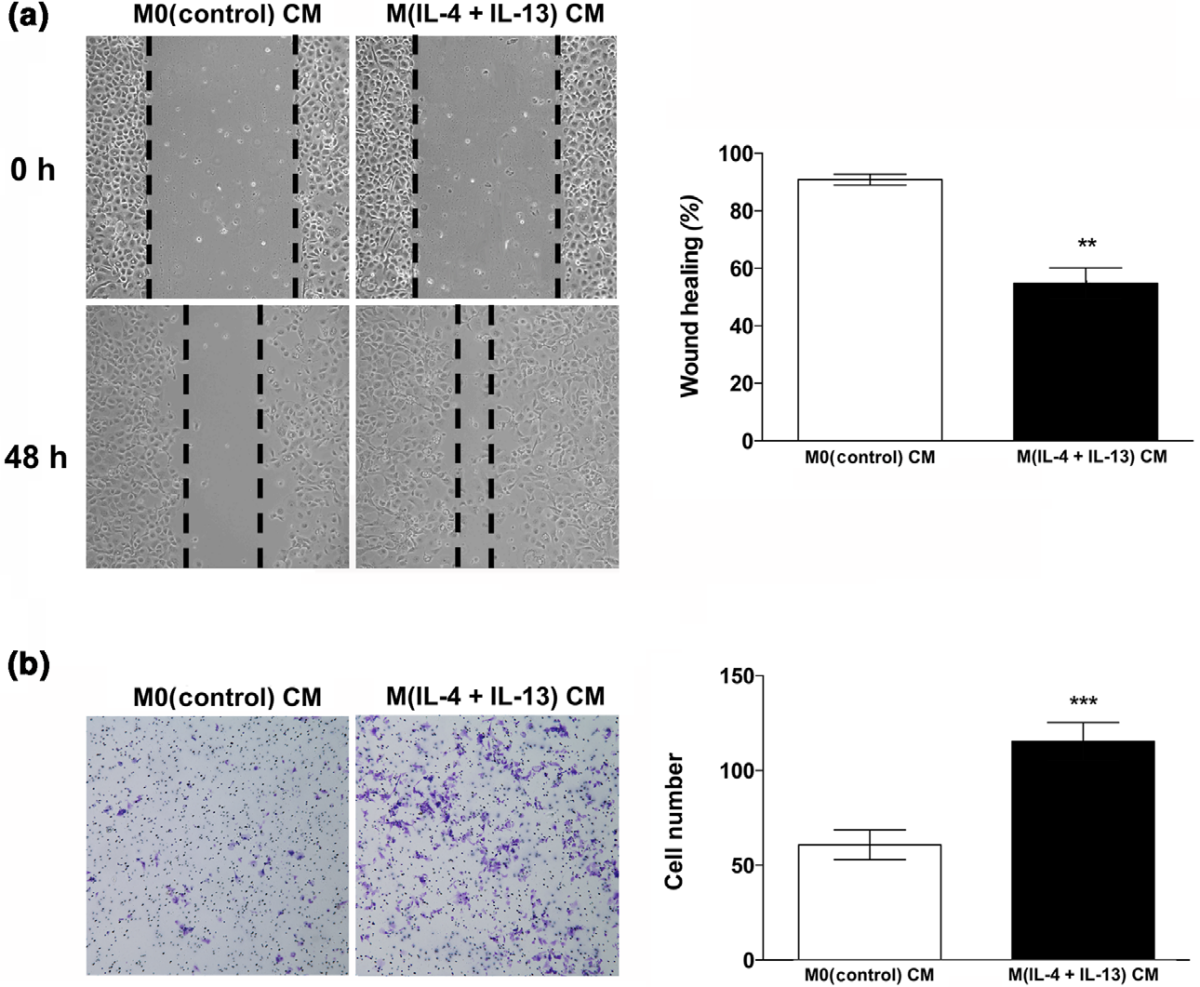
M(IL‐4 + IL‐13) THP‐1 CM increase the migratory behavior of MCF10A cells. **(a)** MCF10A cells were plated in 30‐mm dishes and allowed to reach 100% confluence. The growth medium was changed to THP‐1 CM for 48 h. A pipette tip was utilized to generate a wound (scratch) down the center of the plate. Images of the cells capable of migrating across the wound were captured in triplicate at 100× magnification and the percent wound healing was quantified. **(b)** Transwell assay results and images were captured at 10× magnification. Experiments were repeated three times and the number of cells within a representative 10× field from each experiment was counted. Data within each bar represent triplicate treatments, are presented as mean ± s.e.m. cell number and are expressed as fold change with respect to controls. **P < 0.01, ***P < 0.001 [significantly different from M0(control) THP‐1 CM‐treated cells using a Student’s *t*‐test]. IL, interleukin; CM, conditioned medium.

Considering that tumor cells that have undergone EMT are more likely to metastasize, we next sought to measure the migratory properties of the immortalized MCF10A cells exposed to M0 and M(IL‐4 + IL‐13) THP‐1 CM. First, a simple scratch wound assay revealed that after 48 h, MCF10A cells were more motile when treated with M(IL‐4 + IL‐13) THP‐1 CM (Figure 5a). Next, the MCF10A cells were plated in BD BioCoat control chambers and the cells capable of migrating through the 8‐μm pore toward THP‐1 CM were stained with 10% crystal violet and quantified. We clearly showed that MCF10A cells were significantly more migratory when grown in the presence of M(IL‐4 + IL‐13) THP‐1 CM (Figure 5b).

## Discussion

The PDE model system is an outstanding preclinical model to study immune responses within specialized tissue microenvironments as well as effects on epithelial and stromal responses to stimuli found both endogenously in the breast and exogenously as a result of exposures. Here we demonstrate that we can shift the M1/M2 chemokine profile within human breast tissue PDEs through the addition of IFNγ + LPS or IL‐4 + IL‐13, respectively. Elevated expression levels of M(IFNγ + LPS) markers (*HLADRA* and *CXCL10*) and M(IL‐4 + IL‐13) markers (*CD209* and *CCL18*) are observed in cytokine‐treated tissues. Continuation of PDE studies employed this model system to examine the impact of environmental exposures on breast tissue and measured significant, yet varying responses to BP3 particularly in exposed PDEs. We suspect that the varying responses could be a result of differences in tissue‐related cytokine expression. Relloso *et al*. demonstrated that IL‐4‐induced CD209 expression is reduced in the presence of TGF‐β1 or IL‐10.^30^ Therefore, tissues which appear nonresponsive to BP3 may have higher levels of these cytokines. Alternatively, we would not observe an effect of BP3 on CD209 or CCL18 expression if the cells within PDEs are already polarized toward an M2 phenotype. In either case, our data suggest that variable responses are observed within individuals depending on the timing or microenvironmental cues. The question of whether BP3 exposure results in increased risk may depend not only on the cellular response, but also on whether there are cells with mutations that can respond to these changes. We demonstrated that M2‐related cytokines can induce mesenchymal changes in MCF10A cells. Furthermore, our *ex vivo* culture system reveals that a subset of the PDEs respond to M2‐polarizing cytokines through downregulation of CDH1 suggestive of reduced adhesion and a subset of those responders demonstrated an upregulation of VIM which is reminiscent of EMT changes.

One of the most appealing aspects of employing the PDE model system to study immune‐ and myeloid‐derived changes is the possibility of elucidating how cell signaling within the intact breast tissue responds to cytokines in the growth media. The closest simulation of this biologically relevant system comes from studies which utilize epididymal adipose tissue,^23^ macrophage–epithelial cocultures^17^ and 3D organotypic cocultures.^37^, ^38^ Even so, there are no published findings which reveal the impact of chemokines involved in M2 polarization on normal breast tissues or their role in breast cancer susceptibility. Our data clearly show that polarizing cytokines significantly upregulate accepted M1‐ and M2‐related markers in the myeloid population in a majority of PDE tissues. These findings suggest that tumor tissues or patient‐derived spheroids in explant culture could be an exemplary model system to study how a variety of immunotherapeutic targets could temper the polarization of these resident macrophages toward anti‐inflammatory TAMs because they are paramount effectors in cancer metastasis.^32^

In addition, PDEs are a fitting model system to study the impact of environmental chemicals (endocrine‐disrupting chemicals, EDCs) on cellular alteration and we are hopeful that we can eventually use them to predict risk. Published data suggest that EDCs may impact risk through stromal activation of hormone receptors. BPA is a monomer used in the manufacturing of polycarbonate plastics as well as epoxy resins and is a well‐known environmental endocrine‐disrupting chemical. Interestingly, BPA exposure has been shown to impede the phagocytic activity of peritoneal macrophages and alter TAM phenotypes.^39^, ^40^, ^41^, ^42^ Taken together, these data suggest that BPA‐exposed macrophages may be involved the transformation of ductal carcinoma *in situ* into invasive breast cancer. To this effect, Kim *et al*.^24^ recently demonstrated that BPA increases M2 marker expression in murine macrophage cells and in a coculture system, ductal carcinoma *in situ* cells exposed to BPA‐treated macrophages became significantly more migratory. BP3 is an environmental contaminant on the rise which is used in sunscreens and personal care products to help minimize the damaging effects of ultraviolet radiation. Because of its widespread use and excellent skin absorption, it has been demonstrated that approximately 97% of the people tested have BP3 present in their urine.^43^ Taken together, we extended our PDE studies to investigate how BP3 affects the stromal population within normal breast tissues. Our findings are similar to the studies with BPA and show that a subset of PDEs exposed to BP3 exhibit an increase in markers associated with M2 macrophage polarization. While CD209 and CCL18 expression can be expressed in other myeloid‐derived or stromal cell types, we have demonstrated through direct exposure of macrophages that BP3 can alter these markers in purified human macrophages. Taken together, PDEs from a wide range of patients allow us to study interindividual variation in response to environmental exposures.

Tumor cell metastasis occurs when primary epithelial cells exit their site of origin and colonize at a distant site. Considering that M2 TAM‐mediated EMT is implicated in this process, it is of interest that nonmalignant breast epithelial cells are susceptible to EMT‐associated changes as evidenced by our *ex vivo* and *in vitro* studies. These data suggest that varying genetic backgrounds contribute toward individual differences in breast cancer susceptibility, especially by way of diminished cell–cell contact protein expression. Interestingly, the downregulation of CDH1 and upregulation of VIM observed in response to M2‐polarizing cytokines are similar to EMT changes observed in “active” stromal phenotypes.^44^, ^45^ The “active” phenotype was initially described by Troester *et al*.^45^ and followed up by Roman‐Perez *et al*.^44^ This phenotype displays a gene expression signature indicative of TGF‐β signaling, fibrosis activation, cellular movement and loss of cell adhesion as well as cell–cell contacts. Moreover, these data demonstrate that there is a worse prognosis in estrogen receptor‐positive patients with an “active” stroma. More recent publications have revealed that even normal tissue from women without cancer can be classified into “active” *versus* “inactive” and correlates with adiposity.^46^ Furthermore, the “active” signature can change over time and location in nonaffected breast tissue, suggestive of an immune‐ or stromal‐related change.^47^ Our results suggest that perhaps IL‐4 and IL‐13 either directly or more likely indirectly through polarization of macrophages toward M2 alter the microenvironment to cause the “active” phenotype. Stromal responses likely perpetuate the progression of breast cancer, especially if a premalignant lesion is present, and it is therefore imperative to take advantage of complex model systems that allow us to investigate how varying stimuli can modify both stromal and epithelial responses. We have established that in the normal breast PDE culture system, IL‐4 and IL‐13 or BP3 stimulate chemokines associated with myeloid populations polarized toward M2. Finally, it is important to note that the plasticity of these changes suggests that this is a reversible process.

## Methods

### Cell lines and primary cells

The MCF10A cell line was obtained from American Type Culture Collection (ATCC, Manassas, VA, USA; catalog number CRL 10317), maintained at 37°C in 5% CO_2_ and cultivated in Dulbecco’s modified Eagle’s medium/F12 (Gibco, Grand Island, NY, USA) containing 20 ng mL^−1^ Epidermal Growth Factor (Sigma, St. Louis, MO, USA), 0.5 µg mL^−1^ hydrocortisone (Sigma), 100 ng mL^−1^ cholera toxin (Sigma) and 10 µg mL^−1^ insulin (Sigma) supplemented with 5% horse serum (Gibco) as previously described^48^ and suggested by ATCC. Human THP‐1 monocytes were obtained from ATCC (catalog number TIB 202) and cultured in RPMI (Gibco) supplemented with 10% fetal bovine serum (Gibco) and 0.05 mm β‐mercaptoethanol (Sigma). Peripheral blood mononuclear cells were isolated from the blood of consented patients undergoing elective breast surgery using the SepMate peripheral blood mononuclear cell isolation kit according to the manufacturer’s instructions (Stem Cell Technologies, Vancouver, Canada) and grown in THP‐1 growth medium supplemented with 5 ng mL^−1^ macrophage colony‐stimulating factor (R&D Systems, Minneapolis, MN, USA).

### Treatment and conditioned medium collection

THP‐1 cells were differentiated into M0 macrophages by incubation with 200 ng mL^−1^ phorbol 12‐myristate 13‐acetate (Sigma) for 48 h. Then, the phorbol 12‐myristate 13‐acetate was removed and M0 macrophages were polarized into M2‐like macrophages by supplementing the growth media with 20 ng mL^−1^ of recombinant human IL‐4 protein (R&D Systems Inc.) and 20 ng mL^−1^ of recombinant human IL‐13 protein (R&D Systems Inc.) for 72 h. The cells were collected for RNA isolation and the supernatants from control and IL‐4 + IL‐13 macrophages were centrifuged at 1000*g* to remove cell debris and defined as M0(control) THP‐1 CM and M(IL‐4 + IL‐13)THP‐1 CM, respectively, which were used after addition of 30% fresh complete medium. Pooled human peripheral blood mononuclear cells were left to sit down on the plate THP‐1 media for 7 days and all adherent cells were washed with phosphate‐buffered saline and given either fresh THP‐1 media for M0(control)THP‐1 media with or M(IL‐4 and IL‐13) THP‐1CM to polarize toward M2 for 72 h. Cells were collected for flow cytometry analysis.

### Flow cytometry

Cells were counted and divided into tubes at a concentration of 1 × 10^6^ cells/microcentrifuge tube in a total volume of 0.5 mL. Cells were spun out of media and resuspended in 0.25 mL of Fluorescence‐activated Cell Sorting buffer (1× phosphate‐buffered saline with 0.1% bovine serum albumin). A dilution (125 µL) of CD209 (Thermo Fisher, Waltham, MA, USA; PA5‐78968) was added to each tube for single staining. The cells were then incubated at 4°C with gentle agitation for 1 h. Next, the cells were spun down, washed and resuspended in 250 µL of FACS buffer and a 1:250 dilution of secondary antibody was added to each tube. The cells were incubated at 4°C with gentle agitation for 30 min. The cells were then spun down, washed and resuspended in 100 µL of FACS buffer on ice in the dark. Samples were read using the Amnis FlowSight (Luminex, Seattle, WA, USA). For each sample, 4000 events were recorded both with and without the brightfield. CD209 was read using the 488‐nm excitation laser. Analysis was performed using the IDEAS software (EMD Millipore, Burlington, MA, USA).

### Enzyme‐linked immunosorbent assay

Supernatant was collected from treated PDE cultures, primary macrophages and THP‐1 for the analysis of CCL18 protein secretion using the R&D Systems Quantikine ELISA kit (DCL180B) according to the manufacturer’s instructions (R&D Systems Inc.).

### Patient‐derived explant culture

Fresh breast tissue was obtained from women undergoing breast surgery at Baystate Medical Center, Springfield, Massachusetts, and who were enrolled in the Rays of Hope Center for Breast Research Registry (IRB Baystate Health, Springfield, MA, USA; protocol number 568088). Only benign tissue, as defined by a pathologist, was utilized for PDE studies. The tissue was grossly dissected from the surrounding adipose tissue and placed on SURGIFOAM gelatin sponges (Ferrosan, Sueborg, Denmark) in 30‐mm tissue culture dishes containing 3 mL of medium [phenol red‐free Dulbecco’s modified Eagle’s medium/F12 buffered with 4‐(2‐hydroxyethyl)‐1‐piperazineethanesulfonic acid and NaHCO_3_ from Gibco (Invitrogen, Carlsbad, CA, USA], 5 µg mL^−1^ human insulin, 1× antibiotic/antimycotic (100 U mL^−1^ penicillin/streptomycin and 0.250 µg mL^−1^ amphotericin B), 10 µg mL^−1^ gentamycin from Sigma and 2% charcoal‐stripped fetal bovine serum (Gibco). To stimulate *in situ* macrophage polarization, the medium was supplemented with 10 ng mL^−1^ LPS (Sigma) and 50 ng mL^−1^ IFNγ (Millipore/Sigma) to promote M1 polarization and 20 ng mL^−1^ recombinant human IL‐4 protein (R&D Systems Inc.) and 20 ng mL^−1^ recombinant human IL‐13 protein (R&D Systems Inc.) to promote M2a polarization. PDE cultures were also treated with dimethyl sulfoxide or 30 µm BP3. Following a 72‐h incubation, the tissues and media were flash frozen and stored at −80°C.

### RNA isolation and real‐time PCR analysis

Total RNA from all cell lines and PDEs was extracted in triplicate for each treatment using an acid–phenol extraction procedure,^49^ according to the manufacturer’s instructions (TRIzol, Invitrogen, Carlsbad, CA, USA). Relative expression levels of mRNA were determined by quantitative real‐time PCR using the Mx3005P real‐time PCR system (Agilent, Santa Clara, CA, USA) and all values were normalized to the amplification of an appropriate normalizer gene. Primer sequences for *ACTB,* *CDH1* and *VIM* have been described^50^ and primers shown in Supplementary table 1 were designed to cross exon junctions using Primer BLAST (National Center for Biotechnology information, NCBI; https://www.ncbi.nlm.nih.gov/tools/primer‐blast/). The assays were performed using the 1‐Step Brilliant SYBR Green III QRT‐PCR Master Mix Kit (Agilent) containing 200 nm forward primer, 200 nm reverse primer and 10 ng total RNA. The conditions for complementary DNA synthesis and target mRNA amplification were as follows: 1 cycle of 50°C for 30 min; 1 cycle of 95°C for 10 min and 35 cycles each of 95°C for 30 s, 55°C for 1 min and 72°C for 30 s. Nontemplate controls were included to control for primer dimers and no reverse transcriptase controls were included to control for genomic DNA amplification. Quantitative analysis was carried out as follows: Ct values were assigned for each sample using an automatic threshold level determined by the Mx3000P software. Delta Ct (dCt) values were determined according to the following formula: dCt(target gene)  =  Ct(target gene) – Ct(housekeeping gene). Changes in gene expression are shown as RQ values and calculated using the following formula: RQ = 2^−ddCt^, where ddCt values were calculated as ddCt  =  dCt(sample) – dCt(reference sample).

### Immunohistochemistry

Tissue blocks were sectioned at 4 µm on a graded slide and standard immunohistochemical techniques were carried out as previously described.^51^ For fluorescent immunocytochemistry, primary mouse monoclonal anti‐CD68 antibody (ab955, 1:100; Abcam, Cambridge, UK) and the primary rabbit polyclonal anti‐CD209 antibody (PA5‐78968, 1:1000; Thermo Fisher) were used and immunoreactivity was visualized after incubation with antirabbit secondary antibody coupled with Alexa Fluor‐568 or antimouse secondary antibody coupled with Alexa Fluor‐488 (Molecular Probes, Invitrogen). Slides were cover slipped with VECTASHIELD Mounting Medium for Fluorescence and imaged with a Nikon Eclipse TE2000‐U using NIS Elements Basic Research software (version 4.51). For standard immunocytochemistry, the same mouse monoclonal antibody for CD68 described above and the primary rabbit monoclonal anti‐CD3 antibody (ab16669, 1:100; ABCAM) were used. Immunoreactivity was visualized by incubation with chromogen diaminobenzidine for 5 min. Tissue sections were counterstained with hematoxylin, dehydrated through graded ethanol and xylene and coverslipped. Images were captured with an Olympus BX41 light microscope using SPOT Software version 5.1 (SPOT Imaging Solutions, Detroit, MI, USA).

### Fluorescent immunocytochemistry

MCF10A cells (5 × 10^4^ cells/well) were plated in 8‐well chamber slides and allowed to adhere overnight. The following day M0 or M2 CM were added to the cells and after 72 the media were removed and the assay was carried out as previously described.^50^ Rabbit anti‐VIM (Abcam 92547) was diluted 1:500 and mouse anti‐E‐cadherin (BD Transduction MA1‐10192, Mississauga, ON, Canada) was diluted 1:100 in immunofluorescence buffer [plus 10% goat serum (Sigma) and goat antimouse F(ab′)_2_ (Abcam 6668)] and incubated overnight at 4°C. Unbound primary antibody was removed by washing three times in immunofluorescence buffer and secondary antibody staining incubation and imaging were carried out as described above.

### Migration assays

MCF10A cells were plated to confluence in 6‐well plates and the cell monolayer was scraped in a straight line to create a “scratch” with a p200 pipette tip as previously described.^52^ The cell debris was removed with a phosphate‐buffered saline wash and media were replaced with 2 mL of control or treatment media. The cells were cultured for 24 h and images were captured with a Nikon Eclipse TE‐2000C light microscope using NIS Elements Basic Research software (version 4.51). MCF10A cells were seeded in serum‐free media in Transwell chambers (BD Biosciences) above M0(control) THP‐1 CM or M(IL‐4 + IL‐13) THP‐1 CM. After a 48‐h incubation, chambers were removed and cells were stained with 10% crystal violet. Images were captured with an Olympus BX41 light microscope using SPOT Software 5.1 (SPOT Imaging Solutions, Detroit, MI, USA).

### Statistical analysis

Group means were compared using Student’s *t*‐tests and overall patient responses were analyzed using a Wilcoxon signed‐rank test using GraphPad Prism version 6 (GraphPad Software, La Jolla, CA, USA). A *P* value of <0.05 was considered significant.

## Author Contribution

Kelly J Gregory: Conceptualization; Methodology; Data curation; Formal analysis; Investigation; Methodology; Project administration; Supervision; Writing‐original draft; Writing‐review & editing. Stephanie M Morin: Methodology. Alex Kubosiak: Methodology. Jennifer Ser‐Dolansky: Methodology. Benjamin J Schalet: Tissue Collections. D Joseph Jerry: Writing‐review & editing. Sallie S Schneider: Conceptualization; Supervision; Writing‐review & editing.

## CONFLICT OF INTEREST

There are no conflicts of interest.

## Supporting information

Supplementary figure 1

Supplementary figure 2

Supplementary figure 3

Supplementary figure 4

Supplementary table 1

Supplementary table 2

Supporting information
