# Supplementary figures and images for: The use of patient‐derived breast tissue explants to study macrophage polarization and the effects of environmental chemical exposure
Source: Immunol Cell Biol. 2020 Sep 9;98(10):883–96. doi: 10.1111/imcb.12381 (PMC7754397; doi:10.1111/imcb.12381)

A

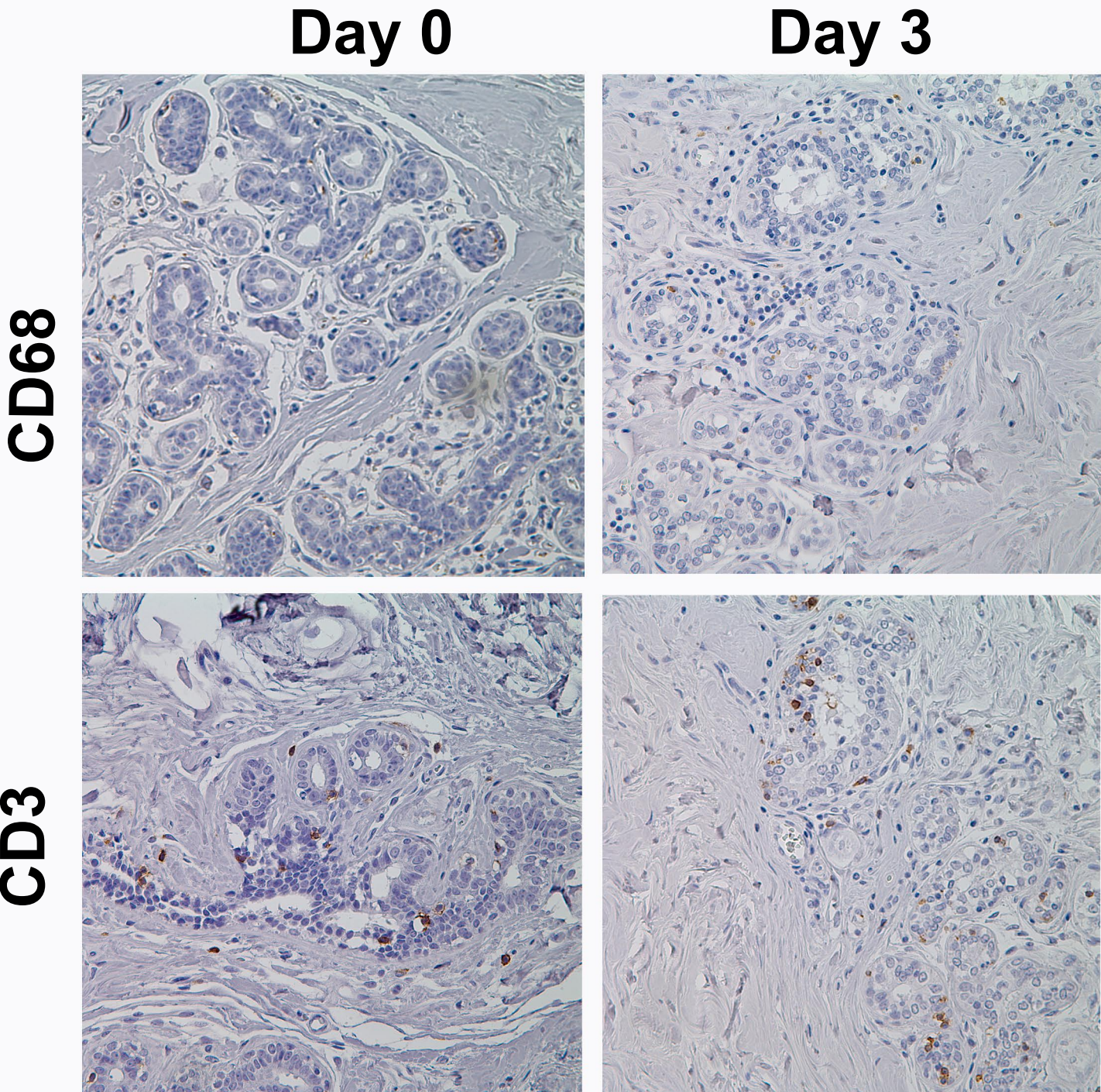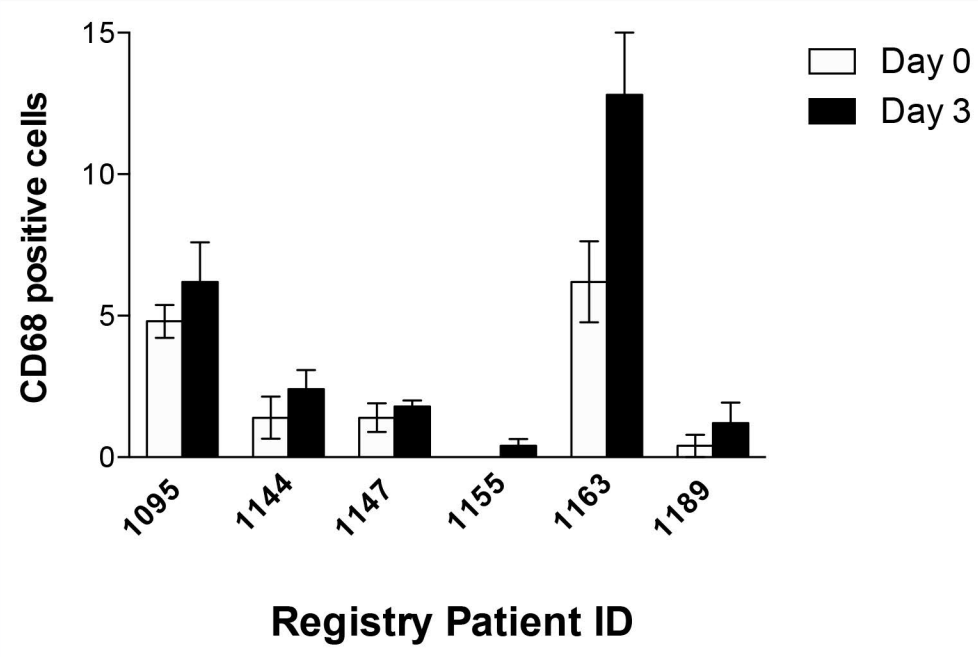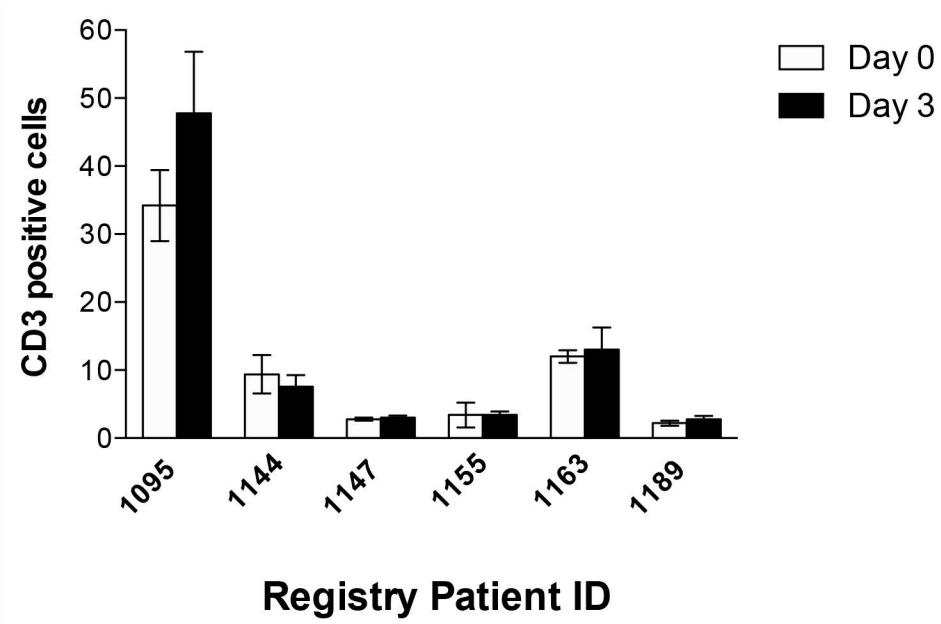

B

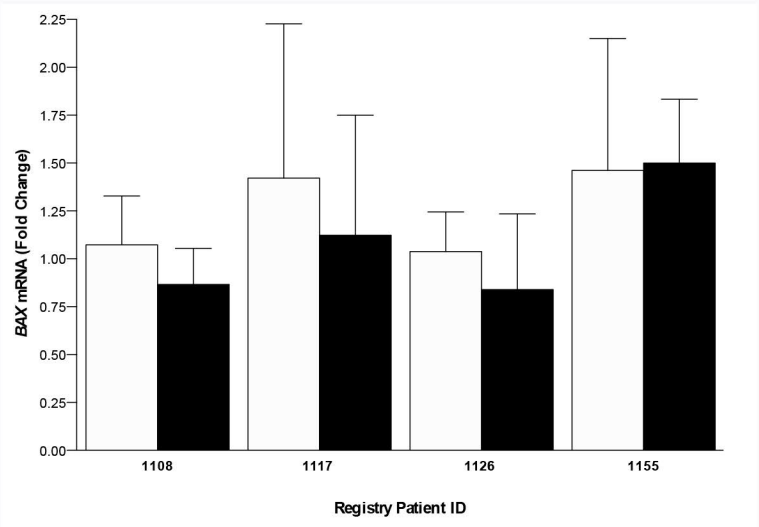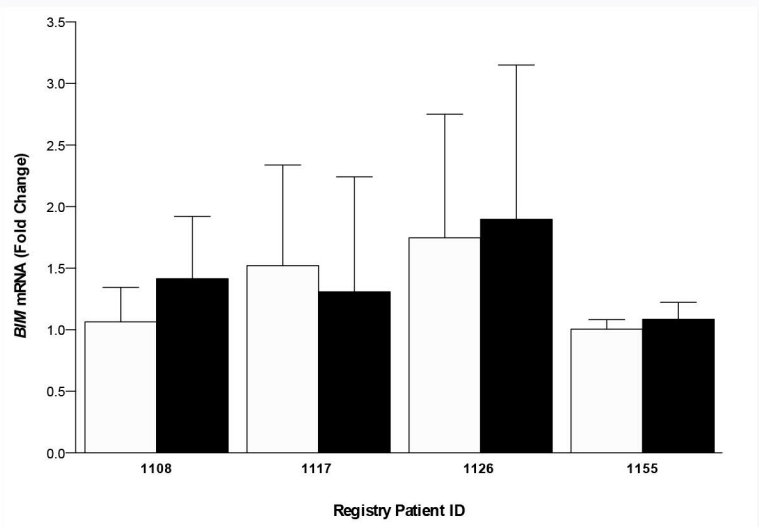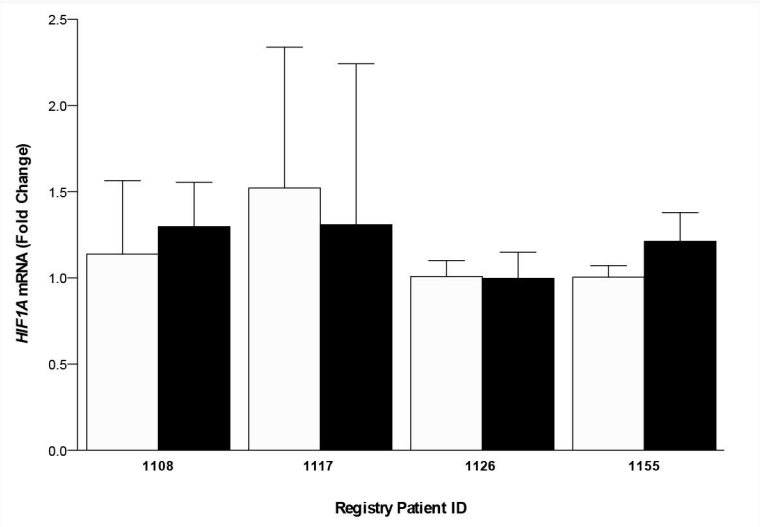

Supplement: Supplementary file 1 — Supplementary figure 1 [file IMCB-98-883-s001.pdf]

A

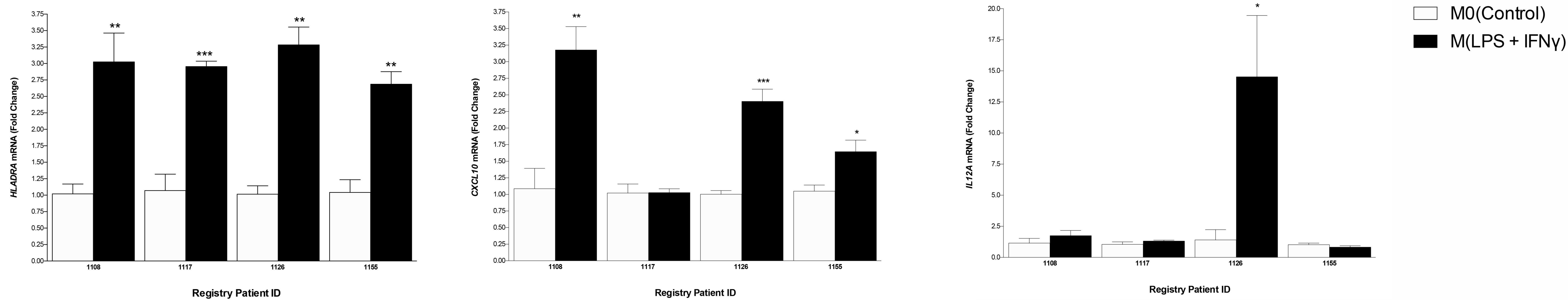

B

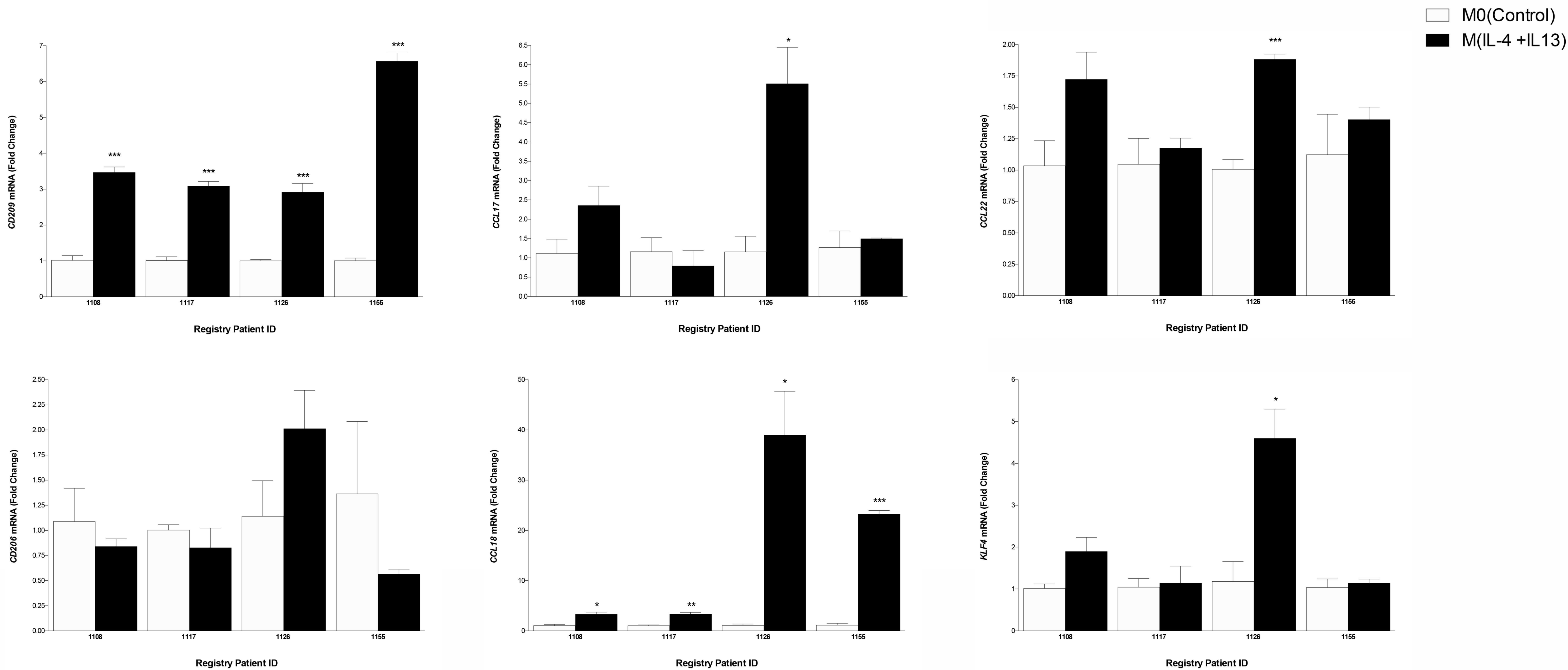

Supplement: Supplementary file 2 — Supplementary figure 2 [file IMCB-98-883-s002.pdf]

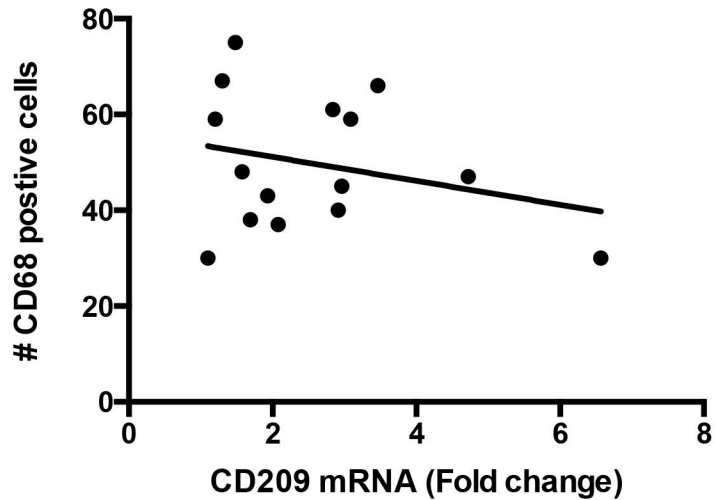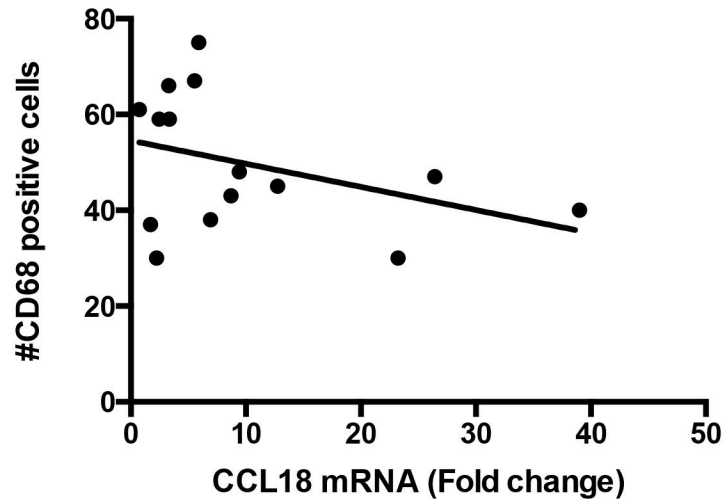

Supplement: Supplementary file 3 — Supplementary figure 3 [file IMCB-98-883-s003.pdf]

**A**

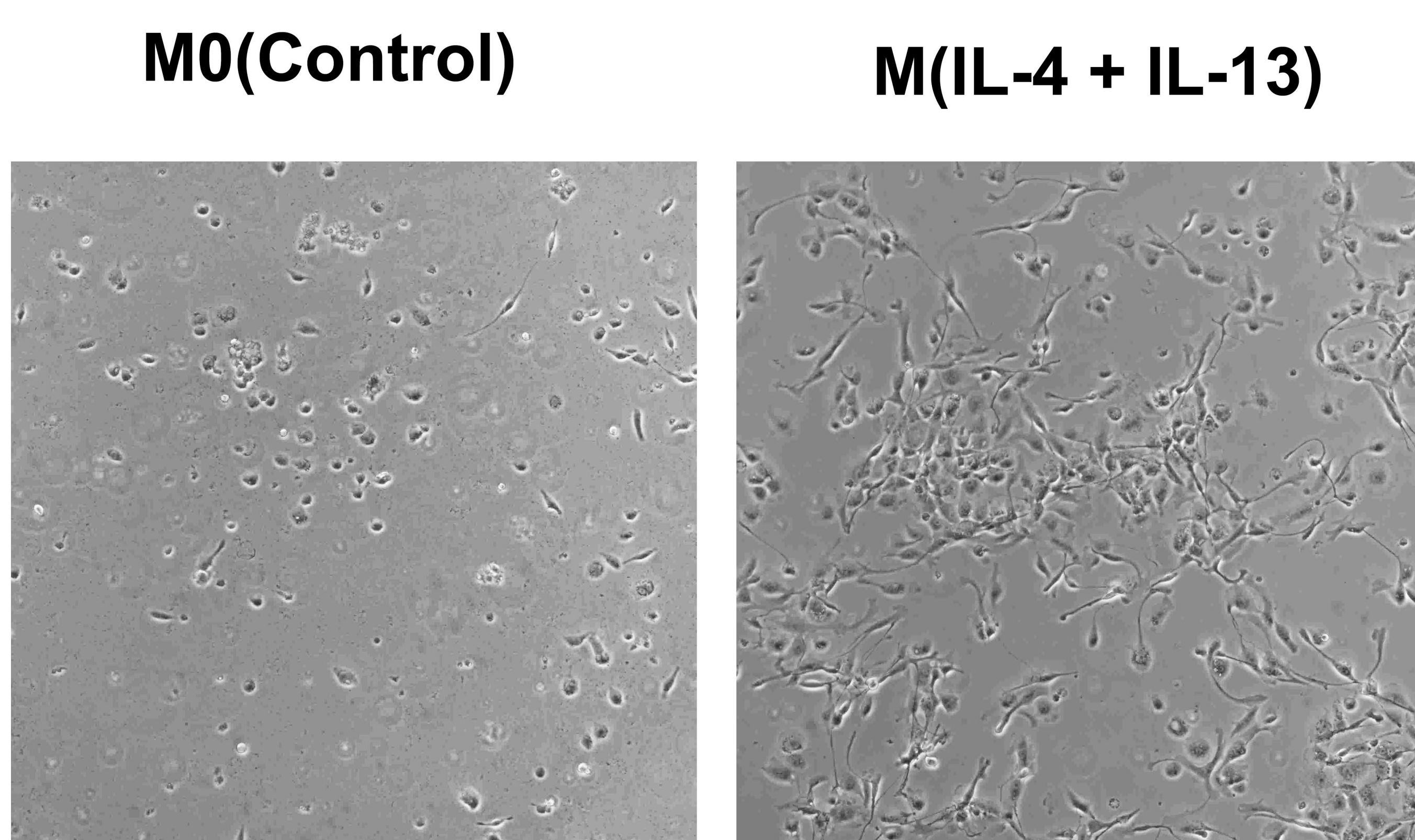

**B**

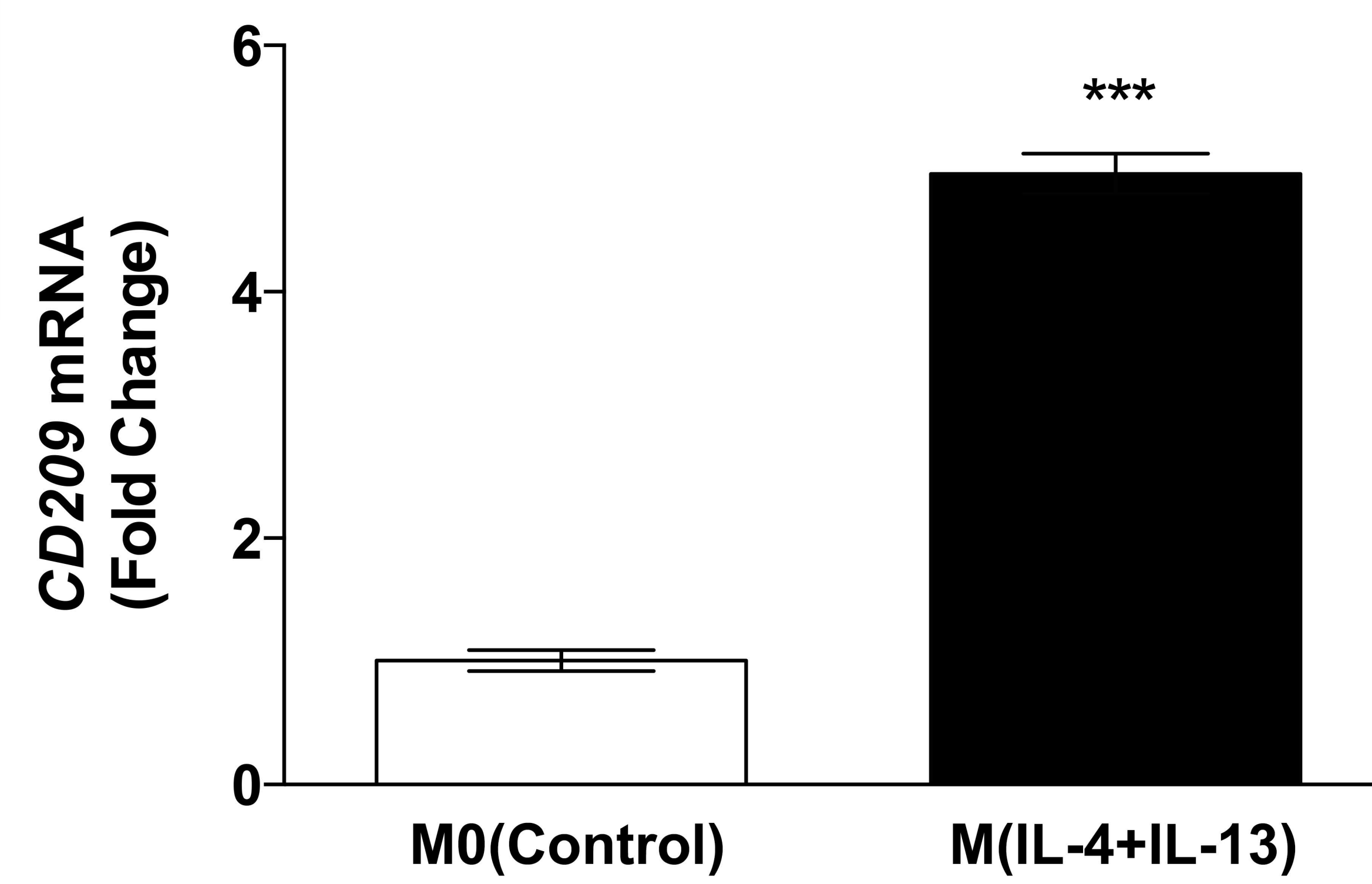

**C**

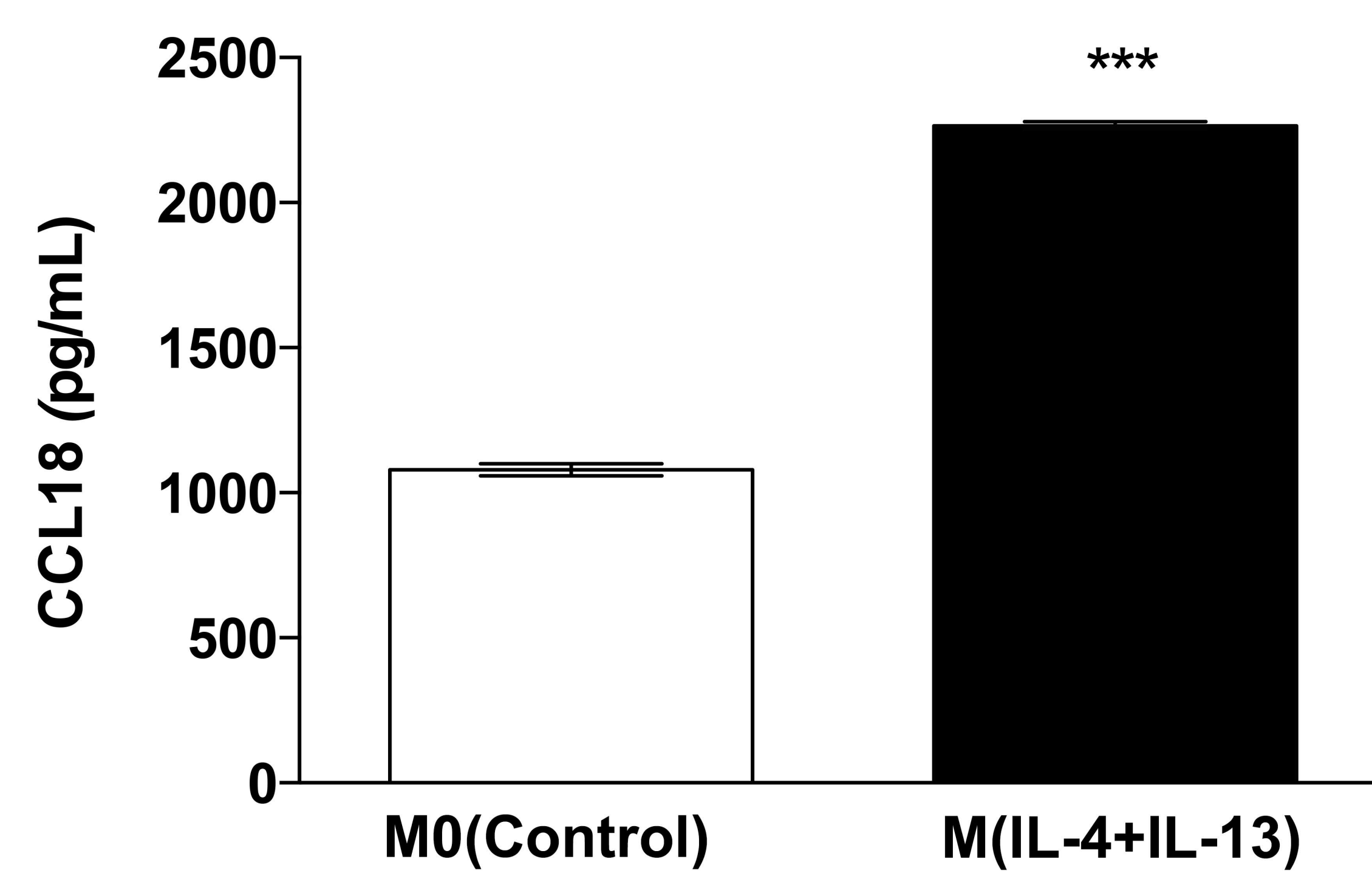

**D**

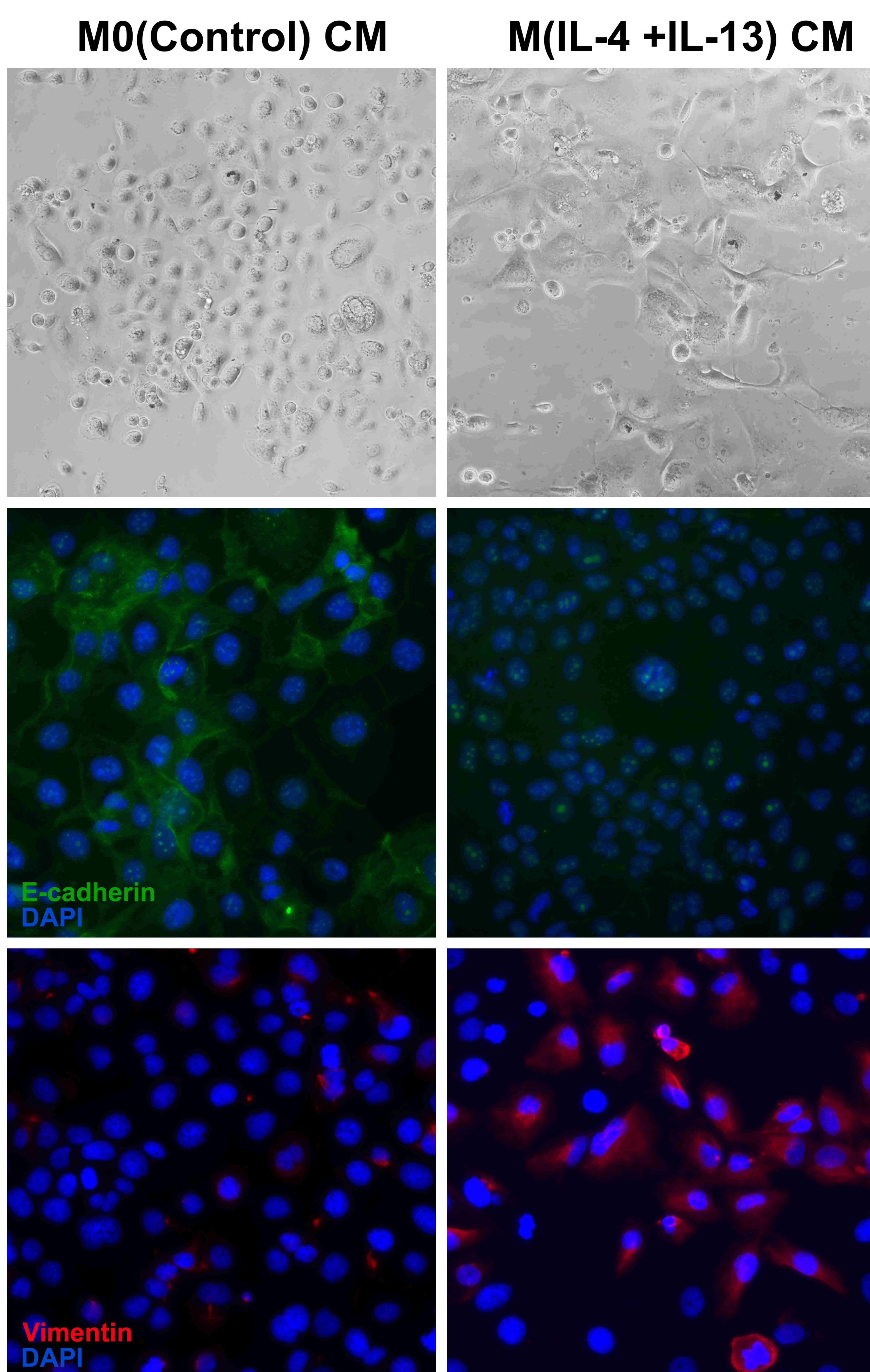

**E**

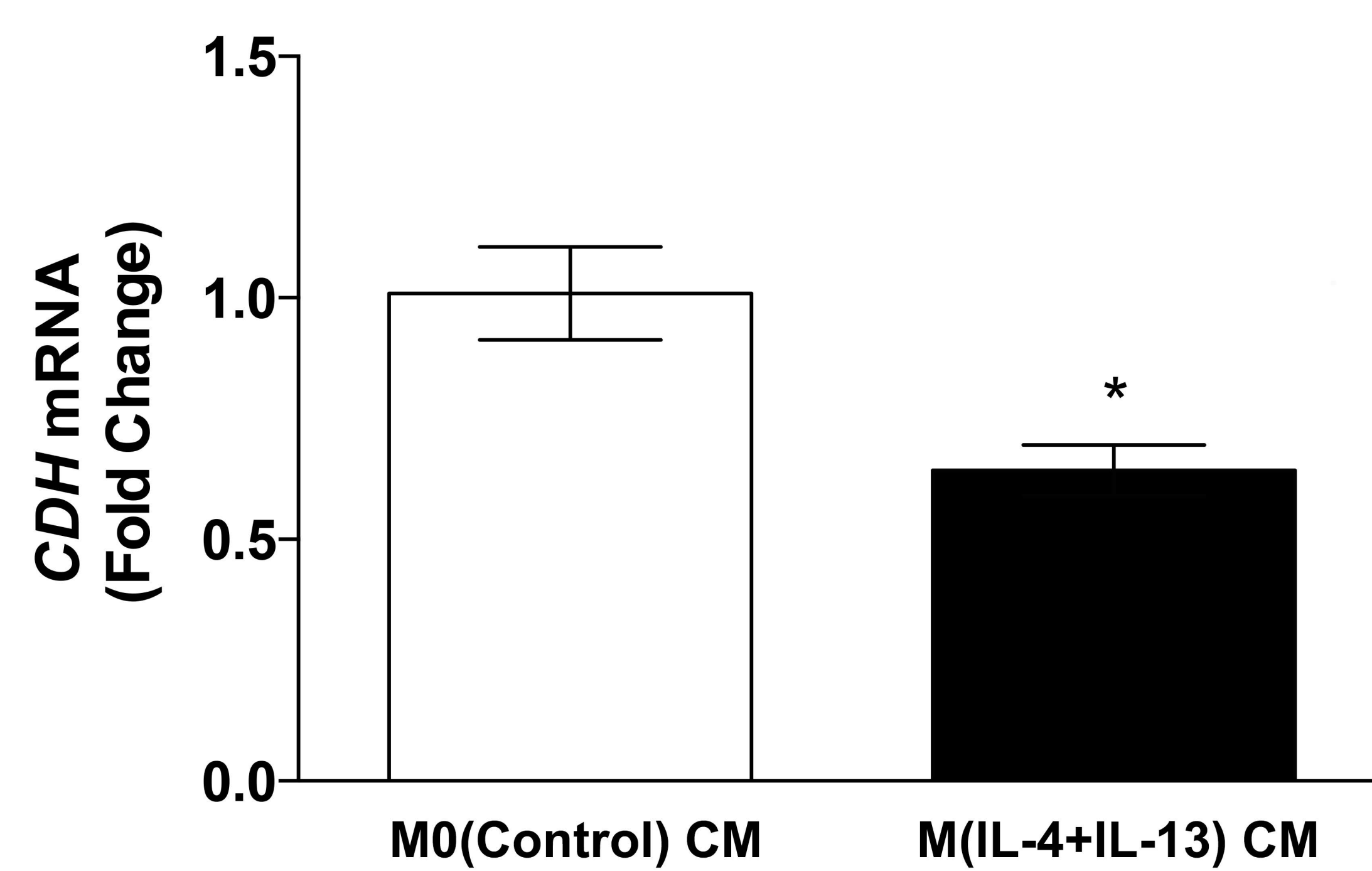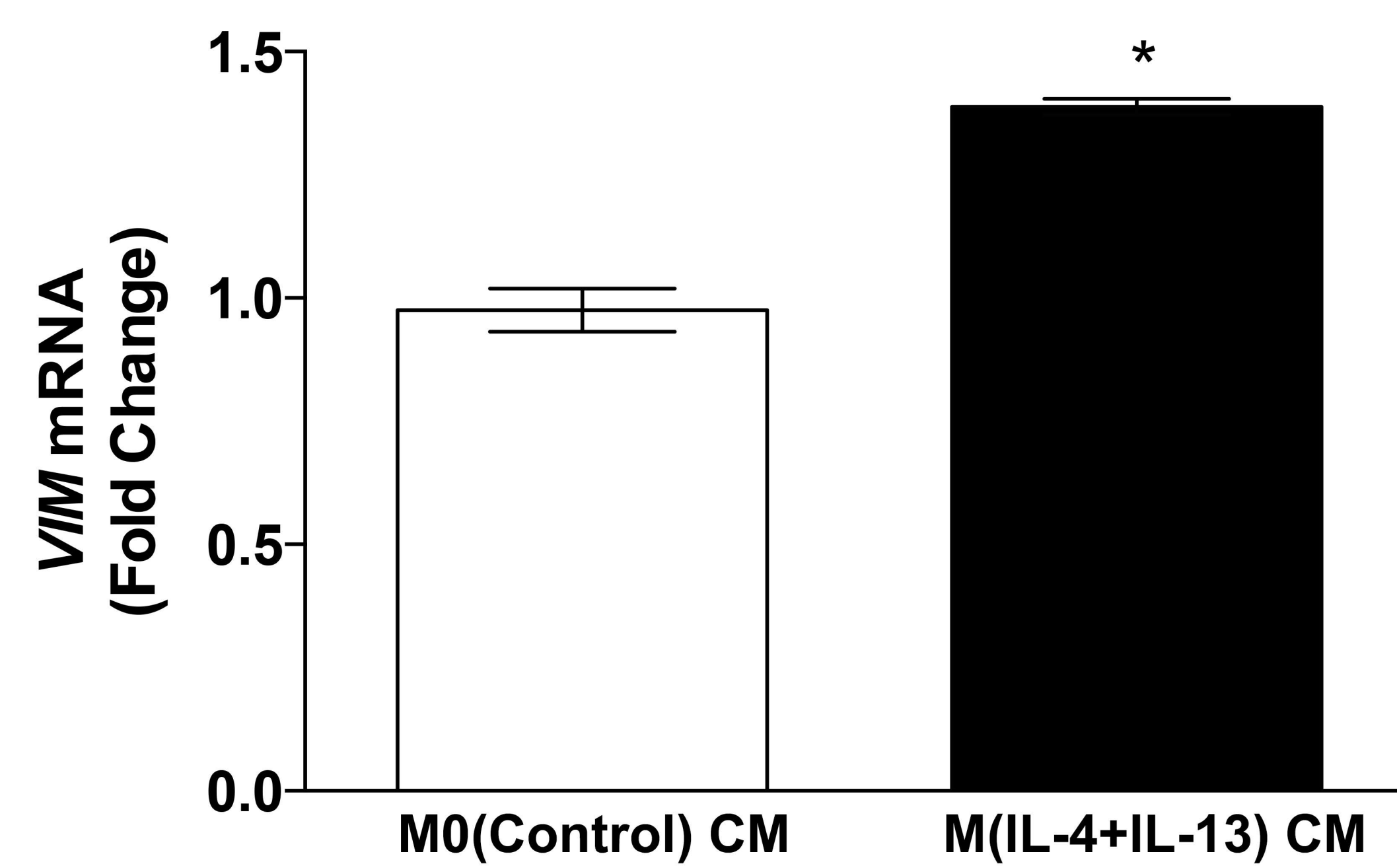

Supplement: Supplementary file 4 — Supplementary figure 4 [file IMCB-98-883-s004.pdf]
