# Supplementary material for: The use of patient‐derived breast tissue explants to study macrophage polarization and the effects of environmental chemical exposure
Source: Immunol Cell Biol. 2020 Sep 9;98(10):883–96. doi: 10.1111/imcb.12381 (PMC7754397; doi:10.1111/imcb.12381)
Supplement: Supplementary file 6 — Supplementary table 2 [file IMCB-98-883-s006.docx]

| **Amplicon** |  | **Primer sequence** | **Amplicon** |  | **Primer sequence** |
| --- | --- | --- | --- | --- | --- |
| *BAX* | forward | 5'-CAAGACCAGGGGTGGTTGG-3' | *CD68* | forward | 5'--GCGGTGGAGTACAATGTGTC-3' |
|  | reverse | 5'-CACTCCCGCCACAAAGAT-3' |  | reverse | 5'-GTGGACAGCTGGTGAAAGAA-3' |
| *BIM* | forward | 5'-ATCTCAGTGCAATGGCTTCC-3' | *CK18* | forward | 5'-CACAGTCTCAGGTTGGA-3' |
|  | reverse | 5'-CAATGCATCTCCACACCAG-3' |  | reverse | 5'-GAGCTGCTCCATCTGTAGGG--3' |
| *CCL2* | forward | 5'-ATTCTCAAACTGAAGCTCGC-3' | *CXCL10* | forward | 5'-CCCACGTTTTCTGAGACATT-3' |
|  | reverse | 5'-CATTGATTGCATCTGGCTGA-3' |  | reverse | 5'-GAGAGGTACTCCTTGAATGC3' |
| *CCL17* | forward | 5'-AATTCAAAACCAGGGTGTCTC-3' | *HIF1A* | forward | 5'-TTTTTCAAGCAGTAGGAATTGGA-3' |
|  | reverse | 5'-GGAATGGCTCCCTTGAAGT-3' |  | reverse | 5'-GTGATGTAGTAGCTGCATGATCG-3' |
| *CCL18* | forward | 5'-AGAAGGAGGCCAGGAGTTGT-3' | *IL12A* | forward | 5'-TGGAGGCCTGTTTACCATTG-3' |
|  | reverse | 5'--GTGGAATCTGCCAGGTA-3' |  | reverse | 5'-CCAGGCAACTCCCATTAGTTA-3' |
| *CCL22* | forward | 5'-GAGTGAAGGAATCCTGGGTA-3' | *KLF4* | forward | 5'-ACCAGGCACTACCGTAAACACA-3' |
|  | reverse | 5'-CCCAAATCCCAGTCTTTAGC-3' |  | reverse | 5'-GGTCCGACCTGGAAAATGCT-3' |
| *CD206* | forward | 5'-TGAAGCCAGGAAAATGGATGG-3' | *NOS2* | forward | 5'-GGGTGCTGTATTTCCTTACGAGCGGGA-3' |
|  | reverse | 5'-TTAGTCAAGGAAGGGTCGGA-3' |  | reverse | 5'-GGTGCTGCTTGTTAGGAGGTCAAGTA-3' |
| *CD209* | forward | 5'-AGGCTTGCTTCGCAGTCATA-3' | *TNFA* | forward | 5'-CCCATTTTGTAGCAAACCCT-3' |
|  | reverse | 5'-CCCCTTGGATTTCAGAGTCA-3' |  | reverse | 5'-TGAGGTACAGGCCCTCTGAT-3' |

**Supplementary Table 1. Primer sequences use for real-time PCR analysis**
