# Supplementary material for: The use of patient‐derived breast tissue explants to study macrophage polarization and the effects of environmental chemical exposure
Source: Immunol Cell Biol. 2020 Sep 9;98(10):883–96. doi: 10.1111/imcb.12381 (PMC7754397; doi:10.1111/imcb.12381)
Supplement: Supplementary file 7 — Supporting information [file IMCB-98-883-s007.docx]

**SUPPLEMENTARY FIGURE CAPTIONS**

**Supplementary figure 1. Immune cell viability in PDEs is not affected by 3 days in culture. (A)** *Left panel,* PDEs were subjected to immunohistochemical analysis, stained for CD68 and CD3 (brown chromagen), and images were captured at 400X. Representative pictures are displayed from each time point which was performed in triplicate samples from each patient. *Right panel,* positive cells were counted (n=5 images/slide) and bars represent bars represent mean ± SEM. **(B)** RNA was harvested from IFN-γ+LPS exposed PDEs (72 hours) and mRNA levels of *BAX, BIM,* and *HIF1A* were analyzed via real-time PCR. All real-time PCR results are from two separate experiments (performed in biological triplicate and technical duplicate) and results were normalized to amplification of *ACTB.* Bars represent mean ± SEM and are expressed as fold change with respect to M0 PDEs.

**Supplementary figure 2.** **Analysis of M(IFN-γ+LPS) or M(IL-4+IL-13) macrophage makers in cytokine stimulated PDEs.** RNA was harvested from cytokine exposed PDEs and mRNA levels of **(A)** M(IFN-γ+LPS) macrophage markers and **(B)** M(IL-4+IL-13) macrophage makers were analyzed via real-time PCR. All real-time PCR results are from two separate experiments (performed in biological triplicate and technical duplicate) and results were normalized to amplification of *CD68* (macrophage marker)*.* Bars represent mean ± SEM and are expressed as fold change with respect to M0 PDEs. * *P* < 0.05, ** *P* < 0.01, *** *P* < 0.001 (significantly different from indicated data set using student’s *t*-test).

**Supplementary figure 3. The number of macrophages within PDE samples is not correlated with M(IL-4+IL-13) marker expression.** PDEs from each patient were subjected to immunohistochemical analysis, stained for CD68, and the total number of CD68-positive cells was quantified. Linear regression analysis of the total counted CD68-positve stained cells and the fold increase in *CD209* or *CCL18* mRNA expression was carried out for all patients described in Figure 1.

**Supplementary figure 4. BP3 increases the expression of CCL18 in human primary macrophages.** RNA was harvested from primary macrophages treated with either vehicle (DMSO) or 30 μM BP3 for 72 hours and the mRNA levels of *CCL18* were measured via real-time PCR. All real-time PCR results are from two separate experiments (performed in biological triplicate and technical duplicate) and results were normalized to amplification of *ACTB*. Bars represent mean ± SEM and are expressed as fold change with respect to vehicle treated macrophages. **P* < 0.05 (significantly different from indicated data set using student’s *t*-test).
